# Transport of dust across the Solar System: Constraints on the spatial origin of individual micrometeorites from cosmic-ray exposure

**DOI:** 10.1098/rsta.2023.0197

**Published:** 2024-06-23

**Authors:** J. Feige, A. Airo, D. Berger, D. Brückner, A. Gärtner, M. Genge, I. Leya, F. Habibi Marekani, L. Hecht, N. Klingner, J. Lachner, X. Li, S. Merchel, J. Nissen, A. B. C. Patzer, S. Peterson, A. Schropp, C. Sager, M. D. Suttle, R. Trappitsch, J. Weinhold

**Affiliations:** ^1^ Department of Solar System, Impacts and Meteorites, Museum für Naturkunde, Leibniz-Institut für Evolutions- und Biodiversitätsforschung, Berlin 10115, Germany; ^2^ Zentrum für Astronomie und Astrophysik, Technische Universität Berlin, Berlin 10623, Germany; ^3^ Center for Electron Microscopy (ZELMI), Technische Universität Berlin, Berlin 10623, Germany; ^4^ Deutsches Elektronen-Synchrotron DESY, Hamburg 22607, Germany; ^5^ Senckenberg Naturhistorische Sammlungen Dresden, Museum für Mineralogie und Geologie, Sektion Mineralogie/Isotope Forensics, Dresden 01109, Germany; ^6^ Department of Earth Science and Engineering, Imperial College London, London SW7 2AZ, UK; ^7^ Space Science and Planetology, Physics Institute, University of Bern, Bern 3012, Switzerland; ^8^ Helmholtz-Zentrum Dresden-Rossendorf, Dresden 01328, Germany; ^9^ Faculty of Physics, Isotope Physics, University of Vienna, Vienna 1090, Austria; ^10^ Forschungs-Neutronenquelle Heinz-Maier-Leibnitz FRM II, Technische Universität München, Garching 85748, Germany; ^11^ Electron Microprobe Laboratory, University of Minnesota, Minneapolis, MN 55455-0153, USA; ^12^ Center for X-ray and Nano Science CXNS, Deutsches Elektronen-Synchrotron DESY, Hamburg 22607, Germany; ^13^ Helmholtz Imaging, Deutsches Elektronen-Synchrotron DESY, Hamburg 22607, Germany; ^14^ School of Physical Sciences, The Open University, Milton Keynes MK7 6AA, UK; ^15^ Dipartimento di Scienze della Terra, Università di Pisa, Pisa 56126, Italy; ^16^ Laboratory for Biological Geochemistry, School of Architecture, Civil & Environmental Engineering, École Polytechnique Fédérale de Lausanne, Lausanne 1015, Switzerland; ^17^ Zentraleinrichtung 3D Technologien (ZE3D), Technische Universität Berlin, Berlin 10623, Germany

**Keywords:** micrometeorites, exposure age, ^26^Al, ^10^Be, AMS

## Abstract

The origin of micrometeorites (MMs) from asteroids and comets is well-established, but the relative contribution from these two classes remains poorly resolved. Likewise, determining the precise origin of individual MMs is an open challenge. Here, cosmic-ray exposure ages are used to resolve the spatial origins of 12 MMs collected from urban areas and Antarctica. Their ^26^Al and ^10^Be concentration, produced during cosmic-ray irradiation in space, were measured by accelerator mass spectrometry. These data are compared to results from a model simulating the transport and irradiation of the MM precursors in space. This model, for the first time, considers a variety of orbits, precursor particle sizes, compositions and densities and incorporates non-isotropic solar and galactic cosmic-ray flux profiles, depth-dependent production rates, as well as spherical evaporation during atmospheric entry. While the origin for six MMs remains ambiguous, two MMs show a preferential tendency towards an origin in the Inner Solar System (Near Earth Objects to the Asteroid Belt) and four towards an origin in the Outer Solar System (Jupiter Family Comets to the Kuiper Belt). These findings challenge the notion that dust originating from the Outer Solar System is unlikely to survive long-term transport and delivery to the terrestrial planets.

This article is part of the theme issue ‘Dust in the Solar System and beyond’.

## 1. Introduction

Solid extraterrestrial material is accreted by the Earth at a rate between 2,000 and 100,000 tons per year. This range in amount is based on data from the collection of space-borne material and terrestrial observations ([1] and references therein). Satellite-based measurements of the meteoroid mass distribution in space have shown the mass flux to peak for particles with a diameter of 220 μm [2]. Cosmic dust particles (melted or unmelted) reaching the Earth’s surface with diameters between ~30 μm and 1 mm are defined as micrometeorites (MMs) [3].

The peak temperature experienced by the MM progenitors during atmospheric entry largely determines whether the particle remains unmelted, whether it melts or whether it evaporates entirely. The peak temperature increases with an increase in (i) entry speed that largely ranges from 11 to 72 km s^−1^ [4], (ii) entry angle, (iii) particle mass and (iv) density [3,5,6]. Besides the entry peak temperature, the particle composition and the heating duration during atmospheric entry determine the degree of melting and evaporation [7].

Unmelted MMs are visually difficult to distinguish from terrestrial rock fragments, while MMs that were subject to melting during atmospheric entry solidify as spheres, also referred to as cosmic spherules. The classification of cosmic spherules is based on their composition where 97% are silicate-rich (S-type), 2% are iron-rich (I-type) and 1% are magnetic-bearing glass (G-type) [3,8]. Among S-type MMs, different atmospheric peak temperatures are thought to lead to different quench textures, i.e. the formation of porphyritic olivine (Po), barred olivine (Bo), cryptocrystalline (Cc) and glass spherules (V) [9].

As is the case for meteorites, a central question for MMs is their origin, i.e. their parent body. Although it is generally assumed that most MMs originate from asteroids and comets, with minor amounts deriving from lunar and planetary debris (e.g. [3]), the following major issues make it difficult to determine how much material reaches Earth from different types of parent bodies: (i) the number of comet-derived particles reaching Earth’s upper atmosphere is underestimated based on MMs found on Earth’s surface. This is because comet-derived particles usually enter the atmosphere at higher speeds and contain more volatiles than asteroid-derived material, which makes them more likely to fully evaporate (e.g. [10]). (ii) The process of melting and partial evaporation during atmospheric entry overprints the pre-atmospheric mineralogical information and can greatly alter the composition (e.g. [6]). (iii) Due to their small sizes, MMs usually do not reflect the average composition of their parent body, but relate to specific sub-components such as chondrules, refractory inclusions or matrix in cases where the parent body was chondritic [3,11].

Despite these challenges, two analytical approaches can be used to decipher the origin and parent body of MMs. The most common approach is to measure the elemental and isotopic composition of MMs yielding a genetic origin. For example, the major element compositions of S-type MMs resemble those of ordinary or carbonaceous chondrites (OC and CC, respectively) (e.g. [11]). This finding is further corroborated by oxygen isotope measurements and indicates an asteroidal origin [11]. Porous anhydrous, volatile-rich MMs with high deuterium/hydrogen ratios and ultracarbonaceous MMs are assumed to derive from comets [12–14].

An alternative but rarely applied approach to decipher the origin of MMs is the quantification of cosmogenic nuclides that accumulated during travel in interplanetary space, yielding an approximate heliocentric distance for the particle’s release from its parent body, i.e. its spatial origin (e.g. [15,16]). Once dust particles are separated from their parent body and released into space, they are exposed to solar photon radiation, which causes them to spiral towards the Sun through the Poynting-Robertson drag [17,18]. During their travel, they accumulate cosmogenic nuclides owing to irradiation by solar cosmic rays (SCRs) and galactic cosmic rays (GCRs)—mostly protons and α-particles that induce nuclear reactions with the atomic nuclei present in the dust particle [15,19]. In large objects, such as meteorites, SCRs, with energies of keV up to ~200 MeV, affect only the surface down to a few cm of depth. This results in a depth-dependent production of cosmogenic nuclides, which also applies to small dust particles [15,20]. GCRs, with energies mostly ranging between some 100 MeV and a few GeV penetrate up to metres in larger planetary materials. In addition, high-energy GCR particles produce so-called secondary particles, which are mostly neutrons that can also penetrate deep into the irradiated object. For small particles, the cosmogenic nuclides produced by GCRs are to first order homogeneously distributed due to the high GCR energies [15,21]. Determining the cosmogenic radionuclide concentration of, e.g. ^10^Be, ^14^C, ^26^Al, ^36^Cl, ^41^Ca, ^53^Mn and ^60^Fe, with half-lives of ~1,000 years up to a few million years, of individual MMs can potentially be used to calculate the cosmic-ray exposure duration (i.e. age) that each particle has experienced before reaching Earth and, hence, allows us to derive the approximate heliocentric distance of its origin.

To date, ^26^Al, ^10^Be and ^53^Mn have been the only radionuclides measured in individual MMs [10,22–27]. To our knowledge, there are only a few MMs (59) for which two radionuclides, i.e. ^26^Al with *t*_1/2_=0.717 Myr [28] and ^10^Be with *t*_1/2_=1.39 Myr [29,30], have been measured simultaneously [10,23–27]. While ^10^Be was used to derive exposure ages, the ^26^Al and ^10^Be pair served as an indicator of the progenitor size and the irradiation history [23,27]. The combined use of these two radionuclides provides a constraint on the heliocentric distance at which the MM originated. This is due to the fact that both isotopes have different half-lives and production mechanisms in S-type MMs, where ^26^Al is largely produced by SCRs and ^10^Be is largely produced by GCRs (e.g. [10]). Furthermore, both radionuclides are contained in refractory minerals and, therefore, experience limited degassing during atmospheric entry melting in contrast to cosmogenic or Solar Wind implanted noble gases [16,31,32].

Three major complications need to be overcome when using cosmogenic radionuclides for deducing the heliocentric distance of MM origin: (i) Pre-irradiation: if a MM progenitor particle was originally close to the surface of its parent body before ejection, it can already have accumulated cosmogenic radionuclides [10]. However, the fraction of radionuclides produced from pre-irradiation becomes irrelevant with increasing travel times of the MM progenitor through the Solar System because of the reduction of the corresponding radionuclide content through radioactive decay. (ii) Saturation: the maximal distance to which the origin of a MM can be determined is limited by the travel time it takes to reach an equilibrium between the cosmogenic production and radioactive decay. (iii) Terrestrial age: once deposited on Earth, the concentrations of the cosmogenic radionuclides are reduced exponentially with increasing terrestrial ages and falsely indicate shorter travel times in space. This problem can be solved if the depositional age of the MM is known.

MMs collected from slow-accumulating environments with potentially high depositional ages, such as deep-sea sediments and Antarctic firn and ice, can have terrestrial ages of millions of years (e.g. [11] and references therein). In such old MMs, the radionuclides may no longer be detectable. Fortunately, it was recently reported that MMs can be found in urban areas, particularly on rooftops of buildings [33]. These particles are likely to be less than a few decades old and have negligible terrestrial ages compared to the radionuclide half-lives [33,34]. Hence, they still contain the highest possible amount of radionuclides.

In this study, we provide a detailed investigation into the use of cosmogenic radionuclides in individual MMs to calculate their residence time in interplanetary space and to infer an estimation of their heliocentric distance of origin in the Solar System. For this, we measured ^26^Al and ^10^Be in six MMs collected on urban rooftops with virtually no terrestrial residence times and six MMs from Antarctic moraine sediments with terrestrial ages potentially exceeding 780 kyr [35]. The data are processed with a newly developed model that simulates the transport and irradiation of MM progenitors in space and determines their exposure times and spatial origin for different progenitor sizes and types, distances of origin and different orbits. Furthermore, this model considers three different SCR flux profiles, solar modulation of GCRs and incorporates depth-dependent SCR production rates within the progenitor particle as well as spherical surface evaporation of different degrees from atmospheric entry heating.

## 2. Material and methods

### (a) Sample collection and micrometeorite extraction

The urban MMs were collected from the following rooftops: the Eugene–Paul–Wigner Physics building of the Technische Universtität Berlin (TUB), Germany (TUBR1, TUBR2), the Memorial High School, Eau Claire, WI (SPMM183), the Augsburg College (SPMM357, SPMM308) and an abandoned mall (SPMM523), both in Minneapolis, MN. Accumulated roof sediment was swept and collected followed by sieving to grain sizes <2 mm and magnetic separation using a bar magnet. Additionally, the TUBR samples were autoclaved for sterilization prior to wet sieving, followed by heating to 600°C in a muffle furnace in order to oxidize organic matter and subsequently wet sieved again. All initial MM candidates were hand-picked from the magnetic fraction based on shape, colour and texture using a stereo-microscope (see [34] for detailed identification criteria). Additionally, a selection of anthropogenic iron-oxide spherules was analysed further as potential candidates for I-type MMs (electronic supplementary material, section 1). The Antarctic MMs from Larkman Nunatak (LN) were collected and extracted from the samples according to Genge *et al*. [35].

### (b) Analytical techniques for sample characterization

The weight of individual MMs was measured twice by two different balances, a Mettler-Toledo XPR2U and a Sartorius Supermicro S4 Ultra-Microbalance to a precision of 0.15 and 0.2 μg, respectively. A simple mean taking the weights as independent values was applied. Optical images were taken with a Nikon D5200 camera attached to an Olympus BH2 BHS stereo microscope and a custom three-dimensional printed stack-shot rail system. Scanning electron microscopy (SEM) and energy-dispersive X-ray spectroscopy (EDX) for surface morphology and chemical composition were performed with a JEOL-6610LV at the Museum für Naturkunde, Berlin, Germany. Elemental compositions were measured at 15 kV. Scanning helium ion microscopy at the Helmholtz–Zentrum Dresden–Rossendorf (HZDR), Germany, was performed using a ZEISS ORION NanoFab with a 30 keV He^+^ primary beam focused on the sample with a spot size <1 nm yielding high-resolution surface-sensitive images with a surface depth of a few nm [36,37]. Charge compensation with an electron flood gun makes surface coating obsolete and unnecessary. A focused ion beam (FIB, Helios NanoLab) was used to cut off a thin section of a non-coated MM. Subsequently, using field emission electron probe microanalysis (FE-EPMA, JEOL JXA-8530F), the elemental composition of the cut surface was determined quantitatively using wavelength dispersive X-ray spectrometers at 15 kV with a probe current of 20 nA and a probe diameter of 1 µm. Both measurements were conducted at the Zentraleinrichtung Elektronenmikroskopie (ZELMI), TUB.

A General Electrics Nanotom M micro-computer tomograph (μCT) at TUB was used for generating three-dimensional density distribution images with a voxel resolution of 0.55 μm using the software VGSTUDIO MAX 3.0. A scaled model was produced from Polyamide 12 by using an EOS Formiga P100 (Laser-Powder-Bed-Fusion) with a layer thickness of 0.1 mm and a focus diameter of 0.4 mm. A three-dimensional absorption and elemental analysis was achieved using the Hard X-ray Microprobe of beamline P06 at PETRA III (Deutsches Elektronen-Synchrotron DESY, Hamburg, Germany) using X-ray fluorescence computed tomography (XRFCT). The microprobe endstation is optimized for scanning X-ray microscopy using KB (Kirkpatrick–Baez) mirrors and provided, in this case, a focused X-ray beam with a size of 410 nm (FWHM) both in horizontal and vertical direction at an X-ray energy of *E* = 18 keV. The sample was scanned through the focus in fly-scanning mode with a step size of 1 μm. An ion chamber upstream of the KB mirrors recorded the incident flux for normalization purposes, while a PIPS (Passivated Implanted Planar Silicon) diode downstream of the sample measured the transmitted beam. The XRF signal was detected using a Maia detector system mounted in backscattering geometry upstream of the sample [38]. The XRF spectra were evaluated using GeoPIXE [39] and the tomograms were reconstructed using a maximum-likelihood expectation maximization algorithm. Animations were produced using the volume exploration and presentation tool Drishti [40] as well as the software VGSTUDIO MAX.

For the instrumental neutron activation analysis (INAA), the iron oxide spherules (electronic supplementary material, section 1) were irradiated at the research reactor FRM II (TU München) in the channel of the capsule irradiation system with a highly thermalized neutron flux of 1.1 × 10^14^ cm^−^² s^−1^ for 48 h [41]. To determine short-lived nuclides, irradiation was carried out in the pneumatic tube system for 15 min. The gamma spectroscopy was carried out in the laboratory of the Radiochemistry Munich (RCM, TU München) on three High Purity Germanium (HPGe) detectors (Mirion/Canberra) with a relative efficiency of 28%, 34% and 40%, respectively and an energy resolution of 1.7 keV at 1333 keV. The software package Genie-2000 as well as MULTINAA and *k*_0_-software were used for the multi-element analysis [42].

### (c) Chemical sample preparation and accelerator mass spectrometry measurements

Following Merchel and Herpers [43] and adapting their procedure for small sample sizes, the MMs were digested in a Parr Instrument general purpose acid digestion vessel at 150°C in a mixture of HF/HNO_3_/HClO_4_. After evaporation and dissolution in HCl, 240–370 µg ^27^Al-carrier and 130–150 µg ^9^Be-carrier were added to each sample and processing blanks (one per four samples). Al and Be separation were achieved by ion chromatography, subsequent precipitation and ignition to oxides. Each sample was mixed with Al_2_O_3_:Ag in a 1:2 weight ratio, and BeO:Nb in a 1:6 weight ratio and was pressed into Cu cathodes.

The samples were measured by accelerator mass spectrometry (AMS) at the Vienna Environmental Research Accelerator (VERA) laboratory, Vienna, Austria. For determination of the ^26^Al/^27^Al ratio, AlO^−^ ions were extracted in the AMS ion source [44]. Selective laser photodetachment of anions was used for suppression of the stable isobar ^26^MgO^−^ [44,45]. Typical AlO^−^ currents were ~550 nA. The ^26^Al/^27^Al data were normalized to the TU Munich standard material of known ratios, i.e. EQ12 with (7.1 ± 1.0) × 10^−13^, and EQ11 with (9.7 ± 0.1) × 10^−12^, as well as to the in-house material SMD-Al-11 with (9.66 ± 0.14) × 10^−12^ [46], a secondary standard directly traceable to primary standards described in Merchel and Bremser [47]. For the measurement of the ^10^Be/^9^Be ratios, the stable isobar ^10^B was suppressed by a silicon nitride foil stack placed in front of the ionization chamber [48]. Typical ^9^Be^16^O^−^ currents were ~1 µA. The ^10^Be/^9^Be AMS data were normalized to the HZDR in-house standard SMD-Be-12 with (1.70 ± 0.03) × 10^−12^, which is traceable to NIST 4325 [49,50]. The ^26^Al and ^10^Be concentrations of the initial MM sample were derived by multiplying the AMS ratios—which include a negligible amount of stable Al and Be from the sample—with the amount of Al and Be atoms from the known carrier addition.

### (d) Transport and irradiation of micrometeorite progenitors in space: theoretical approach

Here, we show the basic equations necessary for deriving the trajectories of the MM progenitors within the Solar System and the concurrent production of radionuclides by irradiation from SCRs and GCR with spatially varying fluxes. This approach is described in detail in the electronic supplementary material, section 2. Similar approaches were discussed previously [25,51].

As soon as the MM progenitors are released from their parent body, they are affected by the Poynting–Robertson drag and migrate towards the Sun. Their orbital distance, given in terms of semi-major axis *a*, and the numerical eccentricity *e*, changes with time *t* as [52]:


(2.1) $$\begin{array}{c} \frac{da}{dt}=-\frac{B\left(2+3e^{2}\right)}{a{\left(1-e^{2}\right)}^{3/2}}, \end{array}$$

(2.2) $$\begin{array}{c} \frac{de}{dt}=-\frac{5Be}{2a^{2}{\left(1-e^{2}\right)}^{1/2}}, \end{array}$$

where the parameter *B* depends on the solar luminosity *L*_ʘ_ = 3.83 × 10^26^ W, the speed of light *c*, and the diameter *d* and mass density *ρ* of the particle, i.e.


(2.3) $$\begin{array}{c} B=\frac{3L_{ʘ}}{8\pi c^{2}d\rho }. \end{array}$$

During their travel, the concentration of cosmogenic radionuclides increases according to the time-dependent production rate *P*(*t*), while radioactive decay reduces their concentration. Thus, the quantity *N*(*t*) of these radionuclides changes with time according to:


(2.4) $$\begin{array}{c} \frac{dN\left(t\right)}{dt}=P\left(t\right)-\lambda N\left(t\right), \end{array}$$

where $\lambda =ln2/t_{1/2}$ is the decay constant.

Both the fluxes of SCRs and GCRs vary with heliocentric distance *r* affecting the radionuclide production rates with time. The variation of the SCR flux is not exactly known. It is determined by a power law, being proportional to *r^−α^*, where the exponent *α* may vary between 1 and 3 depending on the SCR flux profile (e.g. [53]). Hence, for SCRs, the production rate scales with


(2.5) $$\begin{array}{c} P\left(r\left(t\right)\right)=P\left(r_{E}\right){\left(\frac{r_{E}}{r\left(t\right)}\right)}^{\alpha }, \end{array}$$

where *P(r*_E_) is the production rate of the cosmogenic radionuclides from SCRs at the Earth’s orbit.

The GCR flux is considered isotropic in interstellar space, i.e. beyond the heliosphere (~120 au). Within our Solar System, the GCR flux is modulated by the Solar Wind and embedded magnetic fields. The gradient of the solar modulation potential is estimated to be 30 MV/au between 1 and 3 au [21]. This gradient changes for the Outer Solar System. Here, we assume a gradient of 30 MV/au up to 15 au, and a gradient of 2.3 MV/au from 15 au out to the heliosphere (electronic supplementary material, section 3). Consequently, and in contrast to the SCR flux, the GCR flux decreases towards the Sun.

In the chemical compositions considered here, i.e. for chondritic precursor materials, the production rate of ^26^Al is dominated by nuclear reactions on Mg, Si and Al, while the production of ^10^Be mainly depends on the O and C content of the irradiated particle [19]. Depth-dependent SCR production rates at 1 au were modelled using a spectrum with a rigidity *R*_0_ = 100 MV in an energy range of up to 400 MeV [15,20,54]. The SCR-derived production rates decrease with increasing particle sizes. Production rates from GCRs were modelled for different solar modulation parameters (0–1100 MV), where 600 MV corresponds to a distance of 3 au [21]. The GCR production rates for small particles (≤1 cm) are nearly independent of the particle sizes (see electronic supplementary material, section 3).

Finally, we consider different degrees of evaporation of the MM progenitor during atmospheric heating (electronic supplementary material, section 4). We assume an endmember scenario of spherical layer evaporation, i.e. from the surface to the centre, where no internal mixing occurs during this process. For comparison to the experimental data, volume-averaged radionuclide concentrations are calculated for each of the final MM sizes.

The above equations are solved analytically for circular, and numerically for elliptical orbits (electronic supplementary material, section 2). This method computes the build-up of ^26^Al and ^10^Be for an MM progenitor being released at a certain spatial distance from the Sun, i.e. the distance of origin. The final radionuclide concentration is defined as the concentration a particle has gained when its aphelion reaches 1 au. For simplification, the following conditions are assumed:

1. The SCR and GCR source flux is constant in time.
2. No pre-irradiation of the particles on the parent body.
3. The orbits lie within the ecliptic, i.e. inclinations *i* = 0°.
4. No gravitational influences from planets.
5. The MM progenitor sizes and shapes remain constant and spherical during travel, respectively.
6. Their chemical compositions are homogenous and represent material of the parent body.
7. No internal mixing during atmospheric melting.

We computed a grid of 210 different cases that consist of (i) progenitor particle diameters of 90, 200, 400, 800, 2,000, 5,000 and 10,000 µm, (ii) initial eccentricities *e* = 0, 0.6, 0.8, 0.9 and 0.99 at their distance of origin, (iii) initial average chemical composition based on average OC and CC (CI) precursor material coupled with average densities of 3.0 and 2.25 g cm^−3^, respectively (table 1) and (iv) SCR flux profiles for *α* = 1, 2 and 3. Depending on the MM progenitor sizes, the final sizes after spherical layer evaporation are 200, 400, 800, 600, 2,000 and 5,000 µm.

**Table 1.** Types, diameters (*d*), weights (*m*), densities (*ρ*) and chemical composition (wt%), if available, of Antarctic (LN) and urban MMs.

sample	type		d (µm)		m (µg)	ρ (g/cm^3^)	O	Mg	Al	Si	Ca	Cr	Mn	Fe	Ni
*urban MMs*															
SPMM183	Bo		204 × 236		15.1	2.9	31.6	21.5	1.6	18.6	0.7	0.2	bdl	25.8	bdl
SPMM308	Cc		486 × 502		190.3	2.9	42.2	19.6	1.7	20.2	1.0	bdl	bdl	15.3	bdl
SPMM357	Bo		214 × 230		16.2	2.7	39.2	19.0	1.2	14.6	0.6	0.5	bdl	24.9	0.8
SPMM523	Bo		423 × 454		150.6	3.5	29.7	19.9	1.5	19.3	1.6	bdl	bdl	28.0	bdl
TUBR1	Cc		161 × 259		16.2	4.7	44.6	14.1	2.2	17.0	1.2	bdl	bdl	20.9	bdl
TUBR2	Cc		170		10.4	4.0	49.5	16.4	2.4	20.4	0.8	bdl	bdl	14.1	bdl
*Antarctic MMs*															
LN1	µPo		239		20.6	2.9	43.6	15.7	1.1	16.7	1.6	0.3	0.2	20.8	1.2
LN2	Po		244		21.8	2.9	43.3	11.2	2.0	19.5	1.9	0.3	0.3	21.5	bdl
LN4	µPo		91		1.2^a^	2.9	39.4	15.7	0.6	18.5	0.2	bdl	bdl	25.6	bdl
LN6	Cc		278		33.9	3.0	49.9	11.0	2.1	21.2	1.3	0.5	0.3	13.7	0.1
LN7	Bo/Cc		230 × 342		27.6	3.1	39.0	14.4	3.2	22.7	1.9	bdl	bdl	18.8	bdl
LN10^b^	Po		199		13.2	3.2	–	–	–	–	–	–	–	–	–
*chondrites*															
OC						3.0	34.1	14.4	1.2	17.6	1.3		0.3	28.2	1.8
CC						2.25	46.5	9.7	0.9	10.5	0.9		0.2	18.2	1.0

*Notes:* Chemical compositions are from exposed interior cross-section spot analyses with FE-EPMA (LN1, LN2, LN6) and surface spot analyses with SEM-EDX (all other MMs), normalized to 100%. The O values derive from analysis calculation to a total of 100%. In addition, assumed densities and major element compositions of chondritic MM progenitors are given for average OC and CC (CI) progenitors [19 and references therein].

^a^ estimated weight, below detection limit (bld).

^b^ no bulk SEM data available.

Bo, barred olivine; Cc, cryptocrystalline; CC, carbonaceous chondrite; CI, Ivuna-type carbonaceous chondrite; OC, ordinary chondrite; µPo, micro porphyritic olivine; Po, porphyritic olivine.

## 3. Results and interpretation

### (a) Sample characterization

**Figure 1.**
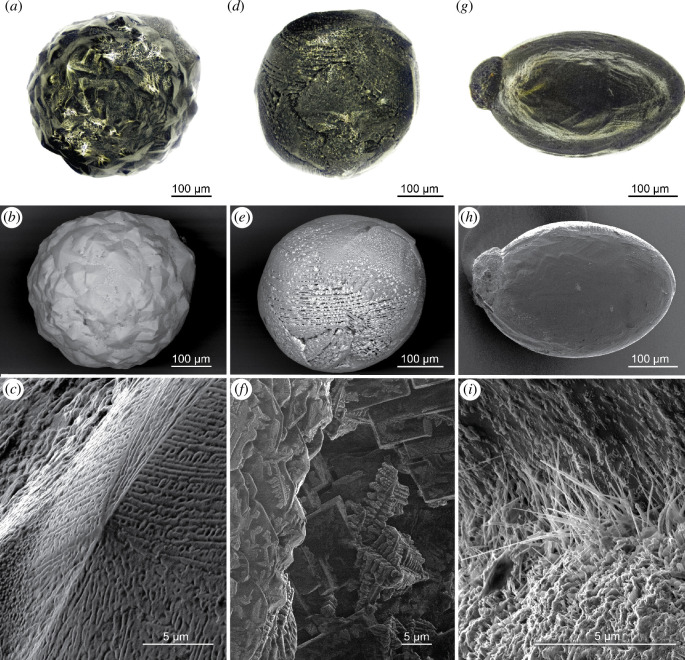
Surface textures of selected urban MMs. (*a–c*) Cc turtleback SPMM308 observed under stereo microscope (*a*), SEM (*b*), and HIM resolving substructures (*c*). (*d–f*) Bo SPMM523 observed under stereo microscope (*d*), SEM (*e*) and HIM resolving overlapping cruciform dendrites of magnetite (*f*). (*g–i*) Cc TUBR1 with protruding metal bead observed under stereo microscope (*g*), and HIM (*h*-*i*) resolving the transition between MM and its metal bead, displaying secondary acicular iron-rich minerals.

The MMs studied here range in diameter from 90 to 500 µm and in weight from 1.2 to 190.3 µg resulting in calculated densities similar to S-type MMs (table 1, see electronic supplementary material, table S1 for anthropogenic spheres). The MMs show a variety of surface textures ranging from porphyritic (with e.g. blocky olivine) over turtle back like (cryptocrystalline with dendritic olivine), striped (barred olivine) to very smooth surfaces of some cryptocrystalline varieties (figure 1, electronic supplementary material, figures S1–S3 including anthropogenic spheres).

**Figure 2.**
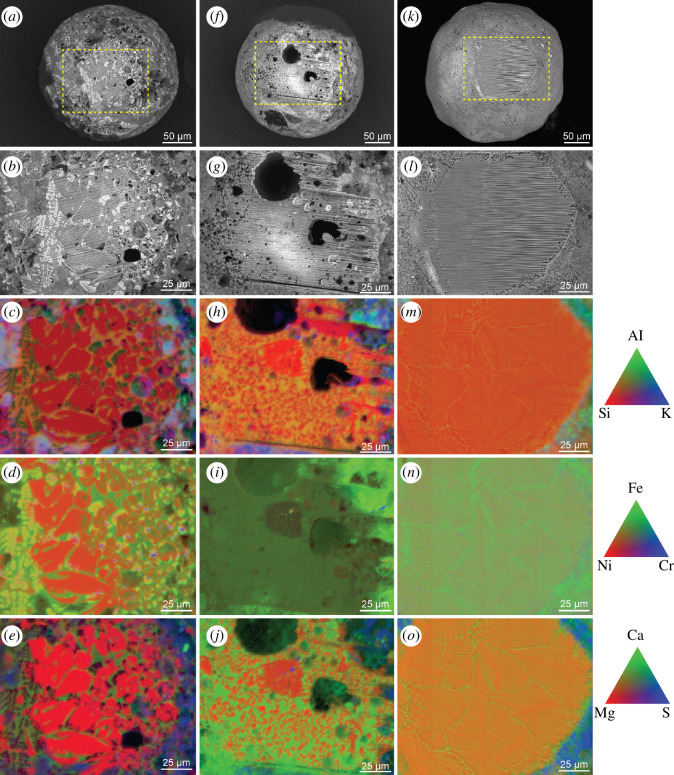
Selected Antarctic MMs and their FIB-exposed surfaces including LN1 (*a–e*), LN2 (*f–j*) and LN6 (*k–o*). The upper two rows show FE-EPMA images and close-ups (yellow frame) of characteristic quench-cooled cosmic spherule textures. The lower three rows show the same cut-out and the results of the FE-EMPA element mapping including the associated non-quantitative element legend on the right side (triangles). LN1 (*a–e*) shows a porphyritic texture composed of olivine phenocrysts and interstitial magnetite. LN2 (*f–j*) shows a micro-porphyritic texture composed of fine-grained olivine phenocrysts embedded in silicate glass, with numerous vesicles preferentially concentrated along one half (right-hand side) of the particle. LN6 (*k–o*) shows a distinctive cryptocrystalline texture termed ‘turtleback’, reflecting a series of interlocking nanocrystalline domains.

To expose the interiors of three MMs (LN1, LN2 and LN6), we cut off thin slices (~15 µm) with a FIB of Ga ions (figure 2, electronic supplementary material, figure S4). The major element compositions from these exposed interior cross-section and surface spot analyses by FE-EPMA (LN1, LN2, LN6) and SEM-EDX (all other MMs), respectively, are shown in table 1. The modal abundance of each phase across the measured surface was considered for calculation of the average composition.

**Figure 3.**
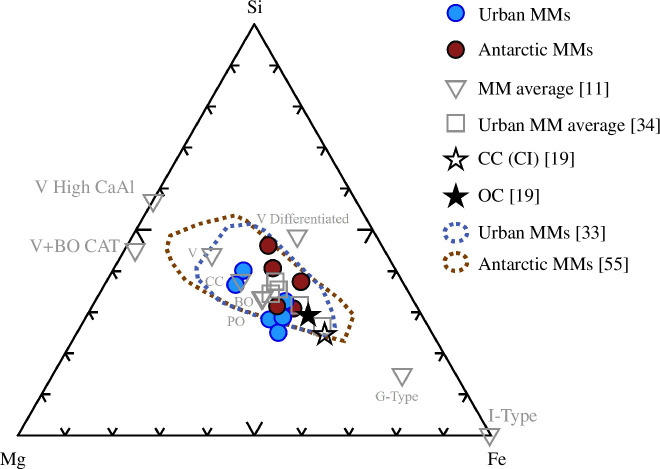
Ternary diagram of elemental compositions (wt%) of urban and Antarctic MMs (table 1) including data from previous studies [33,55] and average compositions of various MM types [11,34], as well as average OC and CC (CI) compositions [19]. Abbreviations: OC = ordinary chondrite, CC = carbonaceous chondrite, CI = Ivuna-type carbonaceous chondrite.

The ternary diagram shows that their major element compositions (Mg, Fe, Si) are comparable to previously analysed S-type MMs from Antarctic deposits and urban MMs recovered from rooftops of buildings (figure 3). INAA conducted on iron-oxide rooftop particles to identify their extra-terrestrial origin yielded negative results because of the low values for Ni (<0.6%; electronic supplementary material, table S1).

The internal textures, as well as surface contamination of LN1, LN2, and LN6 were characterized by FE-EPMA element mapping of the cross-sections exposed with FIB (figure 2). The porphyritic (Po-type) LN1 contains abundant (~50 vol%) equant olivine phenocrysts and cruciform dendrites of magnetite set within an interstitial Ca-Al-rich glass. Olivine crystals include those with skeletal morphologies and are up to 40 µm in length. LN2 is a µPo-type dominated by small (<1–2 µm) equant olivine phenocrysts with an interstitial aluminosilicate glass. Larger (30 µm) enstatite crystals are also present. The spherule also contains subspherical to elliptical vesicles up to 50 µm in diameter. Finally, spherule LN6 is a Cc-type spherule composed of domains of fine olivine-dendrites within an Fe-Ca-Al-rich glass.

**Figure 4.**
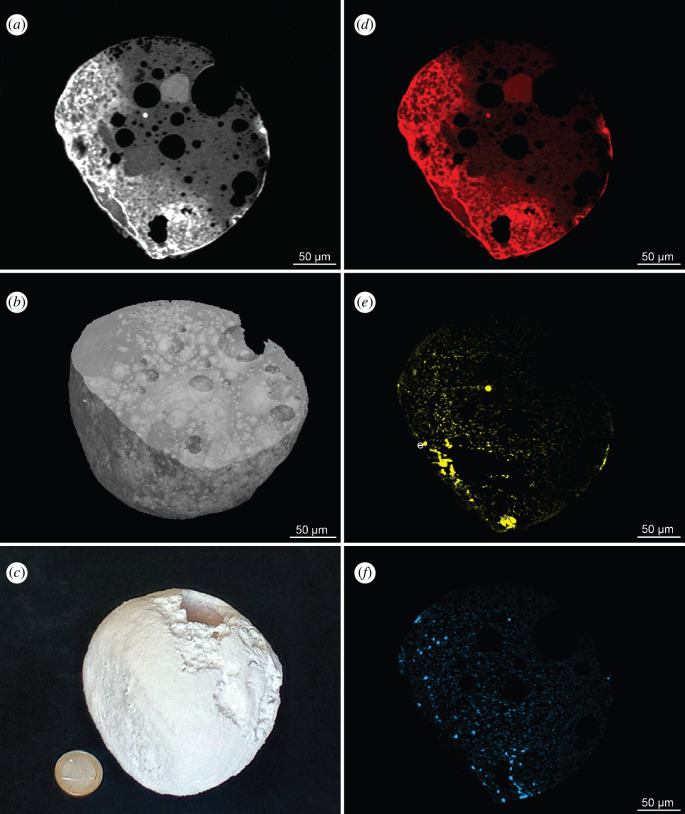
The internal structure of Antarctic MM LN2. (*a*) A slice from a three-dimensional µXRF absorption tomogram showing the internal density structure. (*b*) The distribution of vesicles. (*c*) A scaled three-dimensional model printed from the µCT density distribution is shown in electronic supplementary material, figure S4. (*d*) The Fe, (*e*), Ni and (*f*) Cr distribution in the slice shown in (*a*). This particle has a ‘CumPo’ texture (cumulate porphyritic olivine, as defined in Genge *et al*. [56]). Such textures are produced during atmospheric entry by the density-based segregation of phases, with the high-density olivine crystals settling to the front or leading edge of the MM while the low-density vesicles rose to the back of the particle. These textures indicate an orientated flight (not spinning). Density-separation occurs due to rapid deceleration and could indicate MMs with high orbital eccentricity.

The three-dimensional interior structure and composition has been investigated further for LN2. A density map obtained with µCT and at higher resolution with µXRF indicates the crystalline structure and the distribution of vesicles within LN2 (figure 4). The vesicles derive from degassing during atmospheric passage and heating [57]. During deceleration, higher density phenocrysts of olivine and Fe-Ni-metal concentrate at the front of the MM [3,56]. Such a differentiation is indicated by the density gradient as well as by the distribution of the three elements Fe, Ni and Cr in LN2 (figure 4, electronic supplementary material, media files). Furthermore, the Ni data imply a former Ni-rich metal bead at the front, which likely has been lost.

These data, particularly the three-dimensional mapping of LN2, imply that averages of spot analyses (e.g. EDX) on the surface or exposed interior sections may not represent the overall MM composition. The results of the analysis can differ from the original composition, e.g. by differential evaporation of elements, volatile loss and oxidization. Iron-loss (together with Ni) is one of the most prominent compositional changes during atmospheric entry and can explain the shift from average chondritic towards more Fe-depleted composition. Post-depositional weathering can change the composition even further.

### (b) Experimental results from AMS measurements

All measured ^26^Al/^27^Al and ^10^Be/^9^Be AMS ratios including the carrier were significantly above the processing blank values except for the ^10^Be/^9^Be ratio of the smallest MM, LN4 (diameter: 90 µm). The values of the ^26^Al processing blanks ranged between 1 × 10^−15^ and 3×10^−15^, and a correction of the samples for the processing blank is <7% (except LN4: 29%). The values of the ^10^Be/^9^Be processing blanks were ~2 × 10^−15^. For ^10^Be, a correction of the samples for the processing blank is <17% (except TUBR1: 39%, LN4: 100%). The initial ^26^Al and ^10^Be concentrations in individual MMs and the corresponding specific activities are shown in table 2.

**Table 2.** ^26^Al and ^10^Be concentrations of Antarctic (LN) and urban MMs. Equivalent diameters *d*_eq_ were calculated for non-spherical objects with *d*_eq_=(*d*_maj_ × *d*_min_^2^)^1/3^, where *d*_maj_ and *d*_min_ are major and minor diameters of an assumed elliptical MM shape.

sample	d_eq_ (µm)	^26^Al (10^9^ atoms/g)	^26^Al (dpm/kg)	^10^Be (10^9^ atoms/g)	^10^Be (dpm/kg)
*urban MMs*					
SPMM183	214	65 ± 13	119 ± 24	16.7 ± 1.3	15.9 ± 1.2
SPMM308	491	75.9 ± 5.3	139.5 ± 9.7	14.52 ± 0.41	13.80 ± 0.39
SPMM357	219	121 ± 13	223 ± 23	17.7 ± 1.8	16.8 ± 1.7
SPMM523	433	18.2 ± 1.2	33.5 ± 2.2	6.58 ± 0.24	6.26 ± 0.23
TUBR1	188	22.8 ± 2.7	41.9 ± 5.0	1.59 ± 0.56	1.51 ± 0.53
TUBR2	170	27.2 ± 8.8	500 ± 16	8.0 ± 1.1	7.6 ± 1.1
*Antarctic MMs*					
LN1	239	44.4 ± 7.5	82 ± 14	5.65 ± 0.55	5.37 ± 0.52
LN2	244	23.8 ± 5.5	44 ± 10	5.86 ± 0.47	5.57 ± 0.44
LN4	91	20 ± 17	37 ± 31	0.0 ± 8.1	0.0 ± 7.7
LN6	278	114 ± 11	209 ± 21	16.44 ± 0.69	15.63 ± 0.66
LN7	254	90.3 ± 7.7	166 ± 14	8.24 ± 0.79	7.83 ± 0.75
LN10	199	66 ± 13	122 ± 23	11.1 ± 2.1	10.5 ± 2.0

*Notes:* The 1*σ* uncertainties include the counting statistics from the AMS measurements, the uncertainties from normalization to standard material and the blank corrections. They do not include other uncertainties, e.g. from carrier addition and weighing.

**Figure 5.**
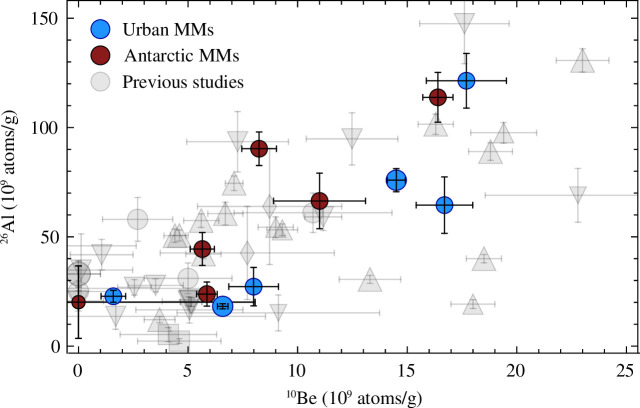
^26^Al and ^10^Be AMS data of urban and Antarctic MMs. Data from previous studies include deep-sea MMs from Raisbeck *et al*. [27] (circles) and Zoppi *et al*. [25] (rectangles), Antarctic and Greenland ice cap MMs from Nishiizumi *et al*. [10] (inverse triangles) and Antarctic MMs from Nishiizumi *et al*. [23] (triangles) and Nishiizumi *et al*. [24] (diamonds). All data from the previous studies used here are given in electronic supplementary material, table S3. The data point sizes relate to the MM sizes.

Similar to previous studies (e.g. [10,27]), we observe a wide range of ^26^Al and ^10^Be concentrations indicating a variety of exposure ages (figure 5). Furthermore, the Antarctic MMs, with terrestrial ages potentially exceeding 780 kyr, do not show significantly lower ^26^Al concentrations compared to ^10^Be as expected from faster radioactive decay. However, a decay-correction shows that terrestrial ages of up to 780 kyr are nevertheless likely for all MMs except LN6 (terrestrial age ≲ 60 kyr) and LN7 (terrestrial age ≲ 200 kyr) (electronic supplementary material, figure S6).

### (c) Transport and irradiation of micrometeorite progenitors in space: theoretical results

The time a particle travels from its location of origin to Earth, as well as the number of orbital revolutions, increases with increasing particle size and decreasing eccentricities (electronic supplementary material, table S2). Hence, larger particles on circular orbits migrate slower towards the Sun than smaller particles on eccentric orbits. The Poynting–Robertson drag reduces mainly the aphelion distance with lesser changes of the perihelion distance, which consequently results in more circular orbits with time (see also [52,58]).

The following notations are used throughout the paper: the heliocentric distance is given in terms of the semimajor axis *a* for eccentric orbits and reduces to radius *r* for circular orbits (*e* = 0). Similarly, the heliocentric distance of origin at time *t*_0_ corresponding to the release of the MM progenitor from the parent object is denoted as *a*_0_ for eccentric and *r*_0_ for circular orbits, respectively.

**Figure 6.**
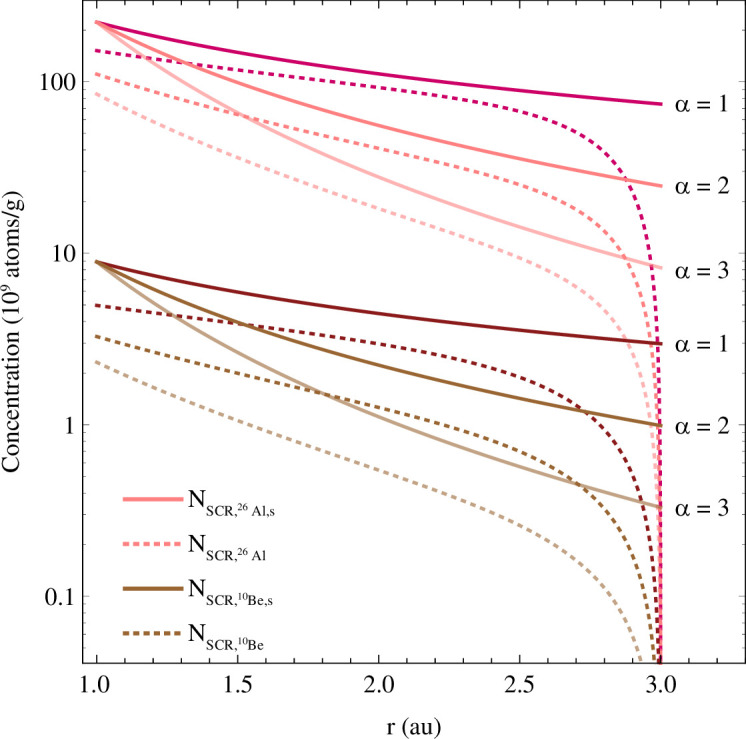
Abundance evolution of saturated (solid lines) and accumulated (dotted lines) SCR-produced ^26^Al and ^10^Be concentrations for an 800 µm diameter MM progenitor with a CC composition and a density of 2.25 g cm^-3^ originating from a distance of 3 au to the Sun and approaching 1 au on a circular spiral orbit (*e* = 0). Three SCR flux profiles are indicated by different *α*-values for solid lines, which correspond to the dashed lines in the same order. The exposure duration for this configuration is 5 Myr.

**Figure 7.**
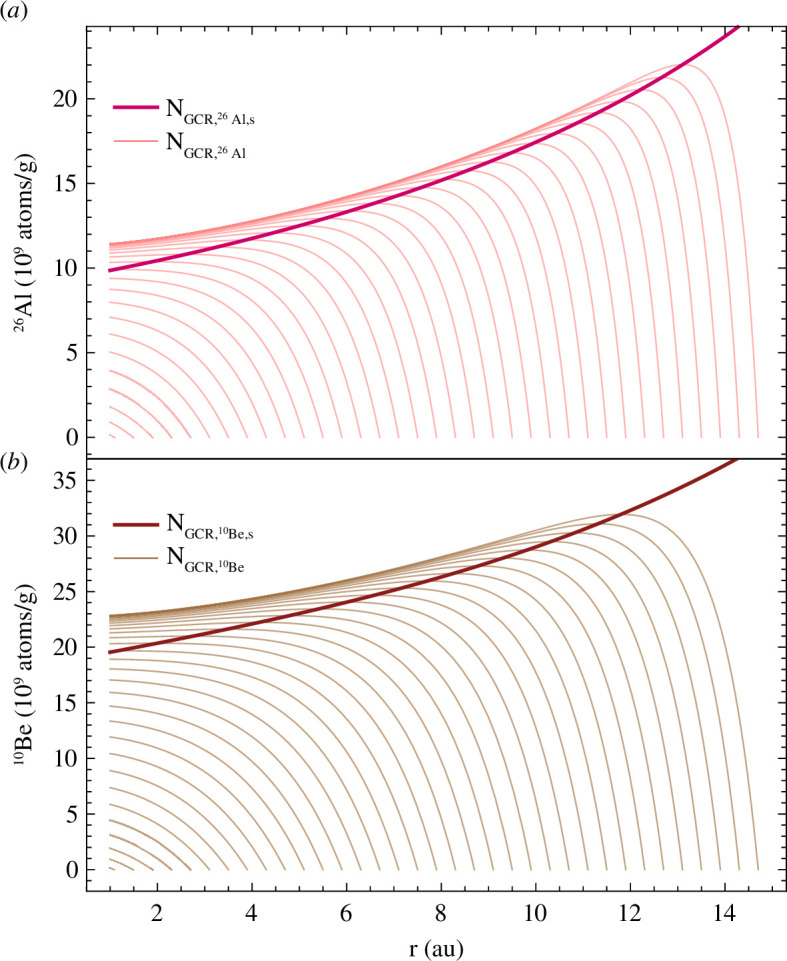
Abundance evolution of saturated (thick lines) and accumulated (thin lines) GCR-produced (*a*) ^26^Al and (*b*) ^10^Be concentrations for a 100 µm diameter MM progenitor with a CC composition and density of 2.25 g cm^-3^ originating from a range of distances between 1.1 and 15 au and approaching 1 au on a circular spiral orbit (*e* = 0).

If particles encountered isotropic SCR and GCR flux densities, the derived ^26^Al and ^10^Be concentrations would reach saturation at some point in time, since the production rates would be temporally constant. However, because of the increasing SCR and decreasing GCR flux with reducing heliocentric distance and the consequential change of the production rates over time, the derived ^26^Al and ^10^Be concentrations never reach saturation (figures 6 and 7). The final SCR-derived concentrations of ^26^Al and ^10^Be at 1 au relate to the solar flux density that depends on the heliocentric distance. Lower values of *α* lead to a larger SCR flux at larger distances, increasing the final ^26^Al and ^10^Be concentrations (figure 6).

**Figure 8.**
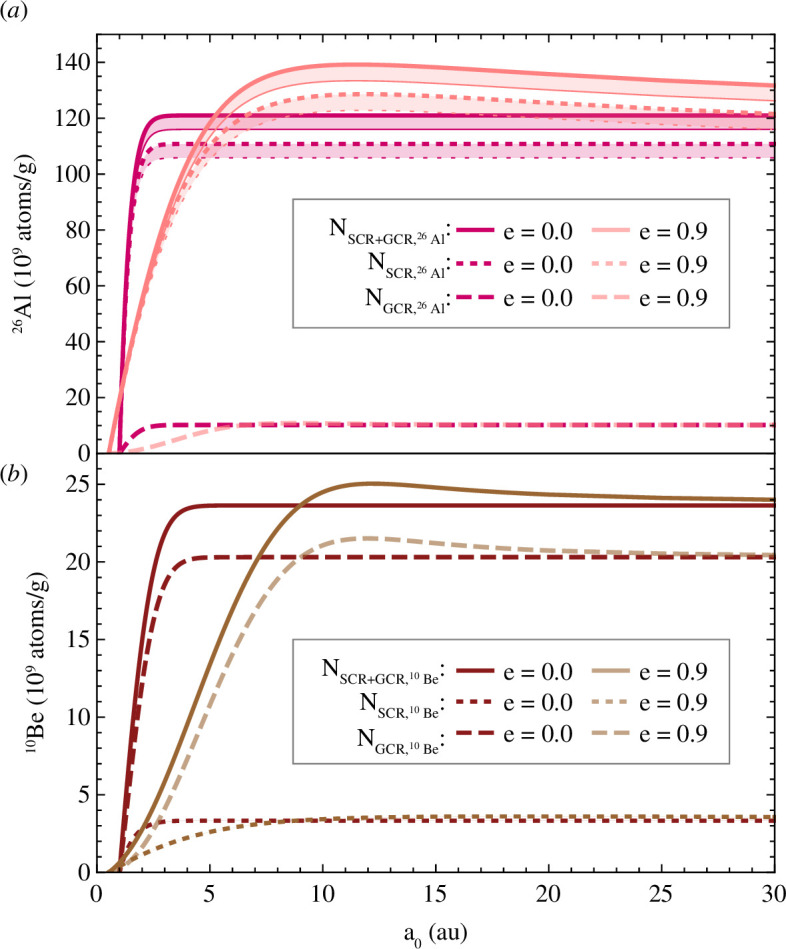
Final concentrations of cosmogenic (*a*) ^26^Al and (*b*) ^10^Be of an 800 µm diameter MM progenitor of CC composition released from distances between 1 and 30 au (*x*-axis). Displayed are the separate SCR (*α* = 2) and GCR contributions and the total amount of radionuclides produced from both components for circular (*e* = 0.0) and elliptical (*e* = 0.9) orbits. Volume-averaged depth-dependent SCR production rates are indicated by concentration bands.

**Figure 9.**
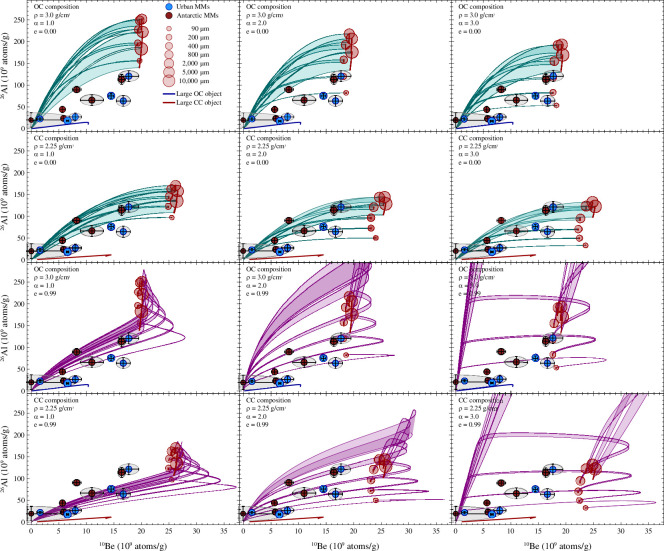
Experimental ^26^Al and ^10^Be data and their positions regarding the modelled concentration bands. Models are shown for orbital eccentricities of *e* = 0.0 and *e* = 0.99, OC and CC progenitor composition, and three SCR flux profiles (*α*). The legend shown in the upper left panel refers to all panels. The red circles show the maximum concentrations corresponding to the upper line of each concentration band. All model results are shown in electronic supplementary material, figures S8 and S9. The blue and red solid lines correspond to the depth-distribution of ^26^Al and ^10^Be in a large (1000 m) object of average OC and CC (CI) composition, respectively, moving on a circular orbit in the Asteroid Belt at 3 au. Abbreviations: OC = ordinary chondrite, CC = carbonaceous chondrite, CI = Ivuna-type carbonaceous chondrite.

For the chemical MM progenitor compositions used here, the SCR irradiation dominates the production of ^26^Al, while ^10^Be is mostly produced by GCRs (figure 8). The accumulated radionuclide content relates to the distance of origin, as shown for GCR-derived ^26^Al and ^10^Be concentrations in figure 7. For circular orbits, the final concentrations at 1 au approach a constant maximum with increasing distance of origin, which differs from the saturation concentration (figures 7 and 8). Particles released on eccentric orbits migrate faster and experience a different cosmic-ray flux profile. Due to the elongated orbits and slower velocities in the vicinity of the aphelion, they spend long periods in regions with enhanced GCR fluxes. Furthermore, the dust particles reach closer distances to the Sun where they experience stronger SCR fluxes, since the model is run until the aphelion distance equals 1 au. Both lead to comparatively enhanced ^26^Al and ^10^Be concentrations for elliptical orbits compared to circular orbits (figure 9). Hypothetically, a particle being released on an eccentric orbit at an infinite distance would travel on a circular orbit within the Solar System, because eccentric orbits become more circular with time. This means the final concentrations of particles on eccentric orbits approach the maximum concentration for circular orbits for large distances of origin.

The depth-dependent production rate of SCR-derived ^26^Al is depicted in figure 8*a*, where the upper and lower line of the band resembles the volume averaged concentrations at the surface and centre of the dust particle, respectively. The depth-dependency of ^10^Be is negligible due to its low production by SCRs (figure 8*b*). Consequently, during atmospheric entry SCR-produced ^26^Al, which is more abundant in the outer layers than in the interior, is lost more easily than ^10^Be. Hence, this process of spherical evaporation changes the pre-atmospheric ^26^Al/^10^Be ratio, which is reflected by subdividing the total (SCR- and GCR-produced) concentration bands of ^26^Al versus ^10^Be into concentration lines for different final MM sizes (electronic supplementary material, figure S7). For this study, we assume an endmember scenario of spherical layer evaporation. A different endmember scenario, in which the radionuclide content is completely mixed and homogenized before evaporation, would result in a concentration line corresponding to the upper line of the concentration band.

### (d) Spatial origins of the urban and Antarctic micrometeorites

We define a match when the error ellipse of the experimental radionuclide data intersects with a concentration band (figure 9). From this, a range for the exposure age as well as the distance of origin is inferred. The given range is, by definition, determined by the measurement uncertainties. Exclusions of improbable solutions in terms of progenitor size and type, as well as parent object constraints by MM textures are discussed below.

In total, 210 concentration bands were calculated including all MM progenitor diameters (seven), orbital eccentricities (five), initial compositions (two) and SCR flux profiles (three). This results in a total of 12 × 210 = 2520 possibilities for the 12 MMs measured in this study. The number of possibilities reduces to 1890 if the concentration bands for progenitor sizes smaller than the size of the MM are excluded. As an example, TUBR1, with a diameter of 188 µm, can only match with concentration bands for MM progenitor sizes of 200 µm and larger, reducing the number of 210 possible concentration bands to 180.

Without constraints, the 12 MMs yield in total 391 matches (20.7%) out of these 1890 possibilities. From the 391 matches, 147 are for OC composition and 244 for CC composition. The results vary for different MM progenitor sizes, SCR flux profiles and eccentricities (electronic supplementary material, figures S10, S11). An example is given in electronic supplementary material, table S4. The smallest MM, LN4, with the lowest radionuclide concentrations and largest uncertainties, matches every concentration band, i.e. all 210 cases. Two MMs with low ^26^Al concentrations and low uncertainties do not match any concentration band (LN2 and SPMM523, table 3). Because SPMM523 is an urban MM, we rule out a significant terrestrial age. Testing the influence of the progenitor density on the final radionuclide concentration implies for circular orbits and the CC production rates used here that densities as low as 0.1 g cm^−3^ are necessary to explain the measured data. Even lower densities of <0.001 g cm^−3^ have been estimated for fluffy cometary aggregates [59]. Another possibility is pre-irradiation on the parent body. Since pre-irradiation was not considered in the model, we show as endmember a hypothetical depth-dependent saturated GCR irradiation profile for a large object (1000 m, figure 9). At this size, the primary and secondary GCRs do not reach the centre of the object, reducing the amount of ^26^Al and ^10^Be in the centre to zero. The ^26^Al concentrations within such an object are below all measured concentrations in the MMs (figure 9). Therefore, the combination of pre-irradiation and post-release irradiation in space could explain the radionuclide concentrations measured in LN2 and SPMM523. Evidence for pre-irradiation of MMs as part of the surfaces of their parent bodies has been found, e.g. in measurement of cosmogenic noble gases [32,60].

**Table 3.** Heliocentric distance probabilities. Number of matches are shown before and after excluding MM progenitor diameters >*d*_p,max_, and removing OC-type results for distances of origin with *a*_0_ < 3.5 au. The probabilities do not sum to 100%, because they are derived from distance ranges that may extend over multiple populations, and the distances between these populations are omitted. Tendencies of origins towards Inner and Outer Solar System, respectively, are printed in bold.

sample	matches (total)	d_p,max_ (µm)	matches (d_i,max_)	NEOs (%)	Mars (%)	AB (%)	IAB (%)	CAB (%)	OAB (%)	JFC (%)	HTC (%)	KB (%)
*urban MMs*												
SPMM183	7	800	4	—	—	25.0	—	—	25.0	—	**25.0**	**50.0**
SPMM308	1	5000	1	—	—	—	—	—	—	—	**100.0**	—
SPMM357	33	800	11	—	—	36.4	9.1	27.3	27.3	36.4	—	9.1
SPMM523	—	—	—	—	—	—	—	—	—	—	—	—
TUBR1	57	800	23	**47.8**	**26.1**	**17.4**	**8.7**	4.3	8.7	4.3	—	—
TUBR2	2	800	2	—	—	—	—	—	—	—	—	**100.0**
*Antarctic MMs*												
LN1	11	800	3	—	33.3	—	—	—	—	—	**66.7**	—
LN2	—	—	—	—	—	—	—	—	—	—	—	—
LN4	210	200	57	85.9	71.9	45.6	43.9	38.6	33.3	24.6	10.5	5.3
LN6	20	2000	10	—	20.0	10.0	10.0	—	—	40.0	10.0	10.0
LN7	25	2000	16	**18.8**	**25.0**	**43.8**	**31.3**	12.5	12.5	—	—	—
LN10	25	800	16	—	6.3	18.8	6.3	12.5	12.5	31.3	12.5	6.3

AB, Asteroid Belt; CAB, Central AB; HTC, Halley-Type Comets; IAB, Inner AB; JFC, Jupiter Family Comets; KB, Kuiper Belt; NEOs, Near-Earth Objects; OAB, Outer AB.

**Figure 10.**
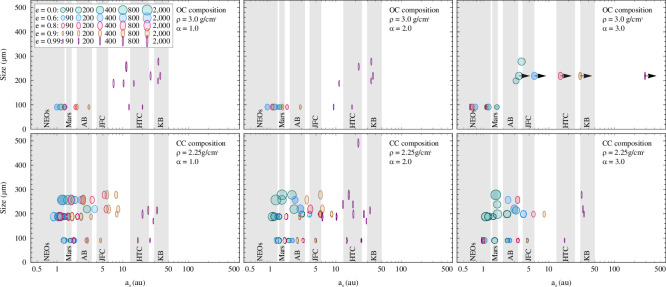
Average distances of origin of urban and Antarctic MMs in terms of their diameters after applying reasonable constraints (see text). The boxed legend showing the MM progenitor diameters (µm) for different orbital numerical eccentricities applies to all panels. The distances of dust-producing regions and populations in the Solar System are indicated as grey bands. Abbreviations: OC = ordinary chondrite, CC = carbonaceous chondrite, NEOs = Near Earth Objects, AB = Asteroid Belt, IAB = Inner AB, CAB = Central AB, OAB = Outer AB, JFC = Jupiter Family Comets, HTC = Halley-Type Comets, KB = Kuiper Belt.

The total number of matches is reduced, i.e. the cosmic-ray exposure ages and distances of origins are better constrained, by applying the atmospheric evaporation criteria from Love and Brownlee [4]. The criteria result in a maximal progenitor size for each MM (table 3, electronic supplementary material, section 4). The remaining results for the average distances of origin determined for each MM are shown in figure 10 (all results including MMs measured in previous studies are given in the electronic supplementary material, figures S12, S13). While CC progenitor models yield a variety of possible distances of origin, OC progenitor models primarily yield origins in the Outer Solar System, which is inconsistent with current consensus on the nature of Outer Solar System bodies (e.g. [13]). Hence, for further consideration, the results for OC progenitor models are limited to distances of origin ≤3.5 au (e.g. [61]).

**Figure 11.**
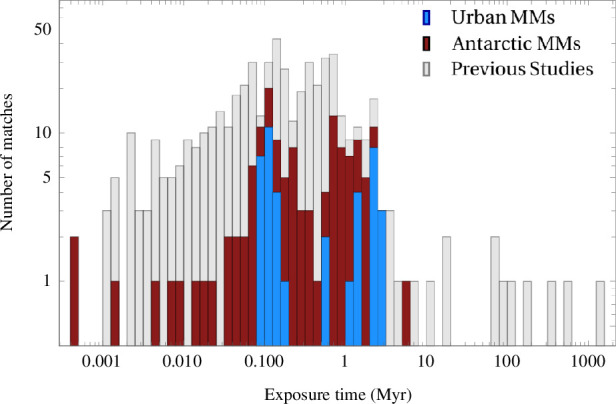
Exposure age histogram. The data include the matches for urban and Antarctic MMs (displayed additive) as well as matches for data from previous studies [10,23–25,27] that yielded 549 matches for maximal progenitor diameters *d*_max_ = 5,000 µm.

The corresponding average exposure ages derived for the here investigated MMs as well as for the MMs measured in previous studies cover a broad range (figure 11). The generally lower uncertainties of our experimental data compared to previous studies lead to a narrower range of exposure ages, which nonetheless reach from close to 0 up to 5.8 Myr, with a mean of 0.7 Myr (median of 0.2 Myr). A similarly broad range of exposure ages has been previously inferred by the measurement of ^10^Be in a set of MMs using the assumption that ^10^Be is only produced by GCRs having a constant flux in space and time [62]. These data were interpreted as indicative of a variety of particle orbits favouring a cometary origin, since a release exclusively in the Asteroid Belt on near-circular orbits would result in a narrower range of exposure times.

The probabilities for distances of origin within different regions and populations in the Solar System are shown in table 3 and are loosely defined by their semimajor axis, and, in the case of Mars, also by the eccentricity. Included are near Earth Objects (NEOs, *a* < 1.3 au), Mars (1.38–1.67 au, *e* = 0), the entire Asteroid Belt (AB, 2.0–3.4 au), the Inner AB (IAB, 2.0–2.5 au), the Central AB (CAB, 2.5–2.82 au), the Outer AB (OAB, 2.82–3.4 au), Jupiter Family Comets (JFCs, 4–6 au), Halley-Type Comets (HTCs, 13–25 au) and the Kuiper Belt (KB, 30–50 au). The probabilities are derived from the ratio of the number of matches within the population to the total number of matches. According to these results, two MMs indicate a tendency towards an origin from the Inner Solar System (TUBR1, LN7) and four MMs show a preferential origin towards the Outer Solar System (SPMM183, SPMM308, TUBR2, LN1). For six MMs, the origin remains unclear, either because origins in the Inner and Outer Solar System are equally likely (SPMM357, LN4, LN6, LN10), or because no distance of origin could be determined with the values used for modelling in the present study (SPMM523, LN2).

Further reduction of total matches for the distances of origin could be achieved, e.g. by relating the MM texture to the nature of the precursor material [9,63]. The majority of the Cc and Po MMs are related to OC parent objects, while Bo and µPo MMs are rather related to CC parent objects [9,63]. If this were that case, three MMs that match only with CC models but relate by texture to OC precursors cannot be assigned to any distance of origin (SPMM308, TUBR1, TUBR2), and the origins of two MMs would reduce to the Outer AB (LN6, LN10). The results for the other MMs relating by texture to CC objects remain similar.

All populations presented here have been discussed as potential sources of interplanetary dust reaching Earth. Dust released within the region of NEOs could derive from near-Earth asteroids or comets, part of which produce the dust trails the Earth’s passes through annually [64]. Mars has recently been proposed as a significant source of dust contributing to the Zodiacal Cloud inventory [65]. However, having a Martian MM among 12 MMs is statistically unlikely, because <1% of MMs are related to differentiated parent objects (e.g. [11]). Numerical models suggested that ~85% of the total dust mass input to Earth originates from JFCs and, furthermore, 20% of the particles released in the KB are able to migrate the entire distance to the Sun [66,67]. Experimental exposure-based methods relying on cosmogenic and Solar Wind implanted noble gases as well as solar energetic particle tracks yield a variety of spatial origins, extending to the KB and beyond (e.g. [15,16,32,68,69]). Our findings, however, rule out the cold KB population with near-circular orbits as a significant dust source since most matches pointing to this region yield highly eccentric orbits (figure 10).

## 4. Discussion and conclusions

Our research provides an important additional method for constraining the origin of MMs. For some MMs, however, no clear assignment to a spatial origin could be found. This is due to uncertainties in the experimental data, as well as unknown parameters underlying the model, which place the following limitations on this method: (i) Experimental instruments such as AMS facilities operate at the detection limit due to the small sizes of the MMs. Fortunately, the AMS efficiency has been significantly improved in recent years, e.g. by installing laser photodetachment systems that greatly improved the measurement efficiency of ^26^Al [44,45] and by advanced detection methods of ^10^Be [48]. (ii) Cosmogenic production rates, especially for the SCR-dominated production of ^26^Al, depend on uncertainties in the SCR spectrum, especially the shape and propagation with distance from the Sun. Here, we thus present models for multiple SCR propagation scenarios (described by the parameter *α*). Half of the GCR energy is contained in galactic α-particles, with uncertain or unknown cross-sections for α-induced reactions. Fortunately, for deriving production rates in MMs, essentially only proton-induced reactions are needed. For those, the cross-sections are well known, especially for ^26^Al and ^10^Be. (iii) Complex irradiation histories of the MMs due to (multiple stages of) pre-irradiation [32,60,70]. (iv) The half-lives of the radionuclides provide a limit to the terrestrial age up to which their concentrations can still be measured with AMS, as the radionuclide concentrations reduce with time.

Additional improvement can be achieved by using a larger set of models to which the experimental data are compared, and which include additional progenitor sizes, initial orbital parameters, compositions and densities to increase statistical significance. For this part, machine learning might provide a significant improvement. Furthermore, measurements of additional radionuclides, such as ^53^Mn with a longer half-life of 3.7 Myr [71], will place further constraints on the identification of spatial origins of MMs. Each method, whether for determining the genetic or the spatial origin of individual MMs, is not conclusive on its own. Apart from addressing the uncertain and unknown parameters that the model depends on, a combination of multiple methods would provide the most reliable results.

Our study provides another substantial contribution to the debate on the survival times of small particles in space (collisional lifetimes), which are derived from dynamical models and which are shorter than their average exposure ages. While the duration required for mutual collisions to completely destroy a dust particle in space is estimated at ~2 × 10^4^ yr [72,73], typical exposure ages, which are also an indicator of the lifetimes, are orders of magnitude larger with 10^5^–10^7^ years [10,23,27,62,74]—a range that is in agreement with our data. This means that even by using a more complex model, we arrive at results similar to previous studies, suggesting either the collisional lifetimes need to be revised or the radionuclide data is misinterpreted [23]. Indeed, a recent study has shown that the effect of mutual collisional may not be as strong as previously thought [75].

Finally, our experimental data are used to estimate the annual influx of ^26^Al and ^10^Be delivered by interplanetary dust to Earth as a fraction of their atmospheric production rates. In the atmosphere, the radionuclides are produced by spallation reactions of secondary cosmic rays with Ar (in case of ^26^Al) and N and O (in case of ^10^Be), yielding average production rates of 1250 ^26^Al atoms yr^−1^ and 6.59 × 10^5 10^Be atoms yr^−1^ [76,77]. The average concentrations measured in the MMs from our study are (57 ± 37) × 10^9^ atoms g^−1 26^Al and (9 ± 6)×10^9^ atoms g^−1 10^Be, respectively. The two radionuclides arrive in an amount of 40,000 (2,000–100,000) tons of extraterrestrial dust annually [1,2]. A distribution over the entire Earth’s surface yields average deposition rates of 450 (8–1853) ^26^Al atoms cm^−2^ yr^−1^ and 73 (1–300) ^10^Be atoms cm^−2^ yr^−1^, corresponding to a fraction of 36% (0.6–148%) for ^26^Al and 0.01% (0.0002–0.045%) for ^10^Be of the atmospheric influx. Thus, while the main ^10^Be contribution to Earth comes from the atmosphere, the interplanetary fraction of ^26^Al may be non-negligible. However, the average fraction derived for the interplanetary ^26^Al influx exceeds previous estimates of 2.8% by Auer *et al*. [76] by an order of magnitude. Furthermore, the upper value of the range exceeds the atmospheric value. This could be due to the following reasons: (i) the radionuclide concentrations measured in MMs with diameters of 90–500 µm are not representative of all particle sizes entering the atmosphere, (ii) the atmospheric ^26^Al production rate derived from measurements of Antarctic firn and ice samples is not representative of the global average production rate and (iii) the upper limit for the dust influx is overestimated. Indeed, the upper limit estimate is uncertain by at least a factor of ~2 [66]. Moreover, the extraterrestrial mass flux inferred from MM collections is significantly lower (down to ~1500 tons yr^−1^ [78]). The latter method mainly includes MM size ranges of ~100–500 µm and, therefore, ignores a certain size fraction of extraterrestrial dust. However, this range includes the main mass peak around dust particle sizes of ~220 µm derived from direct space-borne particle flux measurements [2]. Hence, the true value might be closer to the corresponding dust mass flux of (40,000 ± 20,000) tons yr^−1^ [2].

To summarize, we present new data for the cosmogenic radionuclides ^26^Al and ^10^Be measured in 12 individual urban and Antarctic MMs with sizes of 90–500 µm. A novel interpretation of these data, as well as data from related work, has been performed based on a model for transport and irradiation of progenitor particles in interplanetary space that calculates a variety of orbits, progenitor particle sizes, compositions and densities and incorporates non-isotropic SCR and GCR flux profiles, depth-dependent production rates and spherical evaporation during atmospheric entry. The results show that (i) only a few MMs reach the maximum concentration during their voyage through interplanetary space, which deviates from the saturation concentration, (ii) low experimental measurement uncertainties reduce the amount of possible distances of origin, (iii) models for CC composition agree better with the data than OC models, and (iv) MMs can originate from both the Inner and Outer Solar System.

## Data Availability

The data are made available in the Electronic Supplementary Material [79].
